# Vemurafenib: First-in-Class BRAF-Mutated Inhibitor for the Treatment of Unresectable or Metastatic Melanoma

**DOI:** 10.6004/jadpro.2015.6.4.6

**Published:** 2015-07-01

**Authors:** Lindsay Shelledy, Danielle Roman

**Affiliations:** West Penn Allegheny Oncology Network, Pittsburgh, Pennsylvania

Skin cancer is the most common cancer in the United States, with more than 3.5 million people diagnosed every year (American Cancer Society, 2013). Melanoma is a life-threatening form of skin cancer that occurs due to genetic and environmental factors. The risk factors most associated with malignant melanoma include a family history of melanoma, atypical moles, and previous melanoma. Exposure to ultraviolet radiation and sun sensitivity are additional risk factors for the disease. Although melanoma makes up a small percentage (less than 2%) of all skin cancers, it leads to the majority of all skin cancer–related deaths (American Cancer Society, 2013).

Melanoma develops in stages starting with structurally normal melanocytes or epidermal skin cells. Melanocytes that rapidly multiply form raised or flat lesions, which can develop into abnormal growths that can invade the dermis, ultimately allowing spread to other areas of skin and organs. The initial growth in melanocytes results from atypical activation of the mitogen-activated protein kinase (MAPK) signaling pathway, which may be due to mutations of the BRAF protein in about 50% of melanoma cases (Miller & Mihm, 2006).

Ideally, melanoma is discovered at an early stage, when excision of the cancerous skin cells is recommended with curative intent. In stage II and resectable stage III melanoma, patients may undergo regional lymphadenectomy or removal of local lymph nodes to ensure removal of possible micrometastases. Conflicting evidence exists for the adjuvant use of high-dose interferon alfa-2b (Intron A) therapy in stage II patients with a high risk of relapse (National Cancer Institute [NCI], 2014). Patients with unresectable stage III or stage IV disease can be treated with immunotherapy, signal transduction inhibitors (BRAF and MEK inhibitors), chemotherapy, or palliative local therapy (NCI, 2014).

Survival of stage IV disease is low at 15% to 20% at 5 years and 10% to 15% at 10 years (American Cancer Society, 2013). Therapies are needed to further increase overall survival for patients with later stages of unresectable disease. BRAF protein was identified as a possible target for new therapies, given that mutated forms of the *BRAF* gene lead to unrestricted tumor growth in melanomas.

*BRAF* mutation is most common in patients presenting with skin involvement but without a history of chronic sun-induced damage. Melanomas that arise from mucosal and acral sites are unlikely to present with *BRAF* mutations (Ascierto et al., 2012). Vemurafenib (Zelboraf) is a BRAF kinase inhibitor that blocks tumor growth by hindering cellular proliferation in melanoma cells with the *BRAF* mutation (U.S. Food and Drug Administration [FDA], 2011).

## PHARMACOLOGY

In August 2011, vemurafenib was approved by the FDA for the treatment of metastatic or unresectable melanoma with *BRAF* V600E mutation. This agent was approved along with a new diagnostic test known as Cobas® 4800 BRAF V600 Mutation Test, which helps health-care providers determine whether patients have the *BRAF* V600E mutation. Vemurafenib has not been tested and is not indicated in patients with wild-type *BRAF* melanoma (FDA, 2011).

BRAF has become an important therapeutic target for patients with advanced malignant melanoma. The BRAF protein is normally involved in regulating cell growth. More than 90% of patients presenting with *BRAF* mutation have the V600E-specific mutation. Mutated *BRAF* triggers overactive downstream signaling in the absence of typical growth factors, leading to cell proliferation and survival. Vemurafenib inhibits oncogenic *BRAF* by binding to V600E, which renders the protein inactive, inhibiting downstream proliferation and signaling, ultimately leading to cancer cell apoptosis. Vemurafenib is the first selective inhibitor of mutated *BRAF* and has proven efficacious in several clinical trials (Ascierto et al., 2012).

## CLINICAL TRIALS

The BRIM-3 study was a phase III randomized clinical trial comparing vemurafenib with dacarbazine in 675 treatment-naive patients with *BRAF* V600E-mutated stage IIIC/IV metastatic melanoma. The patients were randomized to receive either dacarbazine (1,000 mg/m₂ intravenously every 3 weeks) or vemurafenib (960 mg orally twice daily). The primary endpoints were overall and progression-free survival. The study also looked at response rate, response duration, and safety as secondary endpoints.

After 6 months of therapy, overall survival was 84% in the vemurafenib group vs. 64% in the dacarbazine group. Progression-free survival evaluated in 549 patients was 5.3 months with vemurafenib and 1.6 months with dacarbazine. Tumor response was evaluated in 439 patients, with overall response rates of 48% and 5% for vemurafenib and dacarbazine, respectively.

Performed after 118 deaths, an interim analysis found that vemurafenib was associated with a relative reduction of 63% in the risk of death and a 74% relative risk reduction of death or disease progression compared with dacarbazine. After review of the interim analysis results by an independent data safety and monitoring board, crossover from dacarbazine to vemurafenib was recommended (Chapman et al., 2011).

A multicenter phase II trial of vemurafenib in patients with previously treated metastatic melanoma with *BRAF* V600 mutation was designed with the primary endpoint of overall response rate and a secondary endpoint of overall survival. A total of 132 patients received vemurafenib 960 mg twice daily until disease progression or unacceptable toxic effects.

The overall response rate was 53%, with 6% of patients achieving a complete response and 47% achieving a partial response. Interestingly, some patients were on therapy for more than 6 months before achieving a response, though most achieved a rapid response to vemurafenib. Median progression-free survival was 6.8 months, and median overall survival was 15.9 months (Sosman et al., 2012).

Vemurafenib monotherapy was recently compared to the combination of dabrafenib plus trametinib in an open-label, randomized, phase III trial of previously untreated patients with unresectable stage IIIc or IV melanoma with BRAF V600E or V600K mutations. A total of 704 patients were randomized 1:1 to receive dabrafenib 150 mg orally twice daily and trametinib 2 mg orally once daily or vemurafenib 960 mg orally twice daily. A preplanned overall survival interim analysis was performed after 77% of the expected events occurred. Overall survival at 12 months was significantly prolonged for the combination therapy group compared to the vemurafenib group (72% vs. 65%; hazard ratio [HR] for death 0.69; 95% confidence interval [CI] = 0.53–0.89; *p* = .005). At this time, the study was stopped for efficacy, and study participants on the vemurafenib arm could cross over to receive combination therapy. Median progression-free survival was 11.4 months in the combination arm and 7.3 months in the vemurafenib arm (HR, 0.56; 95% CI = 0.46–0.69; *p* < .001). The rate of adverse effects was similar between the two groups (Robert et al., 2015).

## ADVERSE EFFECTS

According to the phase II trial, the most commonly reported adverse events included arthralgia, rash, fatigue, alopecia, and photosensitivity. Elevations in liver enzyme levels were observed in several asymptomatic patients. In this study, 45% of patients required dose reductions secondary to rash, arthralgia, liver enzyme elevations, and photosensitivity. Doses were reduced to either 720 mg or 480 mg twice daily.

The most common serious adverse event was development of cutaneous squamous cell carcinoma or keratoacanthoma, which was seen in 26% of patients. The time to development of the first lesion was an average of 8 weeks after initiation of vemurafenib (range, 2–36 weeks). The majority of the lesions were keratoacanthoma or mixed keratoacanthoma type. Dermatologic evaluations were regularly performed, and treatment was surgical excision without the need for dose modification or discontinuation (Sosman et al., 2012).

Common adverse events in the phase III trial were similar to those found in the phase II trial. Adverse effects led to dose interruption or reduction in 38% of patients; however, a few patients experienced grade 3 or 4 adverse effects with vemurafenib. Skin lesions such as cutaneous squamous cell carcinoma, keratoacanthoma, or both developed in 18% of patients taking vemurafenib (Chapman et al., 2011). Similar to the time to appearance in the phase II trial, the lesions first appeared on average of 7 to 8 weeks after vemurafenib initiation. Those 65 years or older, those with a history of skin cancer, and those with chronic sun exposure were identified as being at an increased risk for these lesions (Genentech, 2013).

Peripheral edema, headache, nausea, vomiting, diarrhea, and fever have been recorded in more than 20% of patients receiving vemurafenib. Other serious adverse effects include hypersensitivity reactions, QT prolongation, hepatotoxicity, and ophthalmologic reactions, including uveitis, which may require treatment with steroids and mydriatic eyedrops (Genentech, 2013).

Overall, the most commonly reported adverse events are dermatologic (≥ 30%). One publication assessed three ongoing trials using vemurafenib for *BRAF* mutation–positive advanced melanoma to provide management recommendations for dermatologic adverse effects (Lacouture et al., 2013). This study found that rash and photosensitivity were among the most commonly reported toxicities but were generally manageable with supportive care measures. Most patients with rash were able to maintain full-dose intensity or continue therapy with a modification in dose.

Patients should be counseled to avoid lengthy sun exposure and use protective clothing, sunglasses, and broad-spectrum ultraviolet A/ultra-violet B sunscreen and lip balm (sun protection factor [SPF] ≥ 30). Intervention may be required for macular rash, keratosis-pilaris rash, and palmar-plantar erythrodysesthesia (also known as hand-foot syndrome). Suggested management strategies for these toxicities are listed in Table 1 (Lacouture et al., 2013).

**Table 1:**
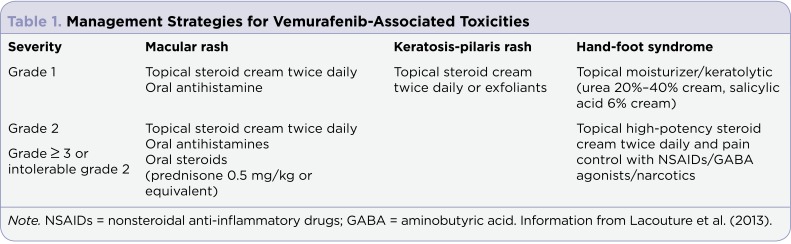
Management Strategies for Vemurafenib-Associated Toxicities

For grade 1 and 2 toxicities, the current dose should be continued and the patient monitored closely for a change in severity. Patients with a toxicity of grade 3 or greater are recommended to have a dose modification, as described in the prescribing information (Genentech, 2013).

If an infection is suspected, a bacterial or viral culture should be obtained. In instances of grade 3 or 4 hand-foot syndrome, treatment should be held until the severity decreases to grade 0 or 1. Reassessment of toxicity after 2 weeks is recommended for all dermatologic toxicities. Dose interruption or discontinuation may be necessary if the reaction worsens or does not improve (Lacouture et al., 2013).

## CLINICAL IMPLICATIONS

Vemurafenib, the first agent to target mutated BRAF, is an option for the treatment of advanced or metastatic melanoma based on favorable results from the BRIM-3 trial. The FDA has subsequently approved two additional agents that target *BRAF*-mutated disease: dabrafenib (Tafinlar) and trametinib (Mekinist). Dabrafenib, which specifically targets and inhibits *BRAF*, similar to vemurafenib, may be associated with less cutaneous squamous cell carcinoma or keratoacanthoma than vemurafenib, although it is associated with more pyrexia. Trametinib inhibits *MEK1* and *MEK2*, which are further downstream of *BRAF*. Single-agent trametinib is associated with lower response rates in treatment-naive patients compared with BRAF inhibitors. According to the National Comprehensive Cancer Network (NCCN), vemurafenib is an option for the treatment of advanced or metastatic melanoma in patients with anticipated clinical deterioration of ≤ 12 weeks (NCCN, 2015). Based on recent phase III trial results, the combination of dabrafenib and trametinib should be considered a preferred first-line therapy option over vemurafenib monotherapy for the majority of patients.

Additional studies have been undertaken to investigate the role of vemurafenib combination therapy. A phase III study evaluated the combination of vemurafenib and cobimetinib, a potent MEK inhibitor, compared with vemurafenib and placebo. The study found the addition of cobimetinib to vemurafenib significantly improved progression-free survival, with some increase in toxicity (Larkin et al., 2014).

## DOSING AND TOXICITY MANAGEMENT

Vemurafenib is available as 240-mg tablets to be taken twice daily without regard to meals. The recommended dosing is 960 mg, or 4 tablets twice daily until disease progression or unacceptable toxicity. A missed dose may be taken up to 4 hours prior to the next scheduled dose.

Dose reduction is indicated with grade 2 or 3 toxicities. Treatment should be interrupted until the toxicity reduces to grade 0 or 1; then vemurafenib should be resumed at a reduced dose. In instances of grade 4 toxicity, vemurafenib should be held until the toxicity returns to grade 0 or 1; then the dose should be resumed at 480 mg twice daily or discontinued permanently.

Due to the risk of cutaneous malignancies associated with the use of vemurafenib, patients should receive dermatologic evaluation before initiating therapy and every 2 months while on therapy. In addition to dermatologic evaluation, liver function tests, electrolytes, and a periodic electrocardiogram should be monitored, as listed in Table 2 (Genentech, 2013).

**Table 2:**
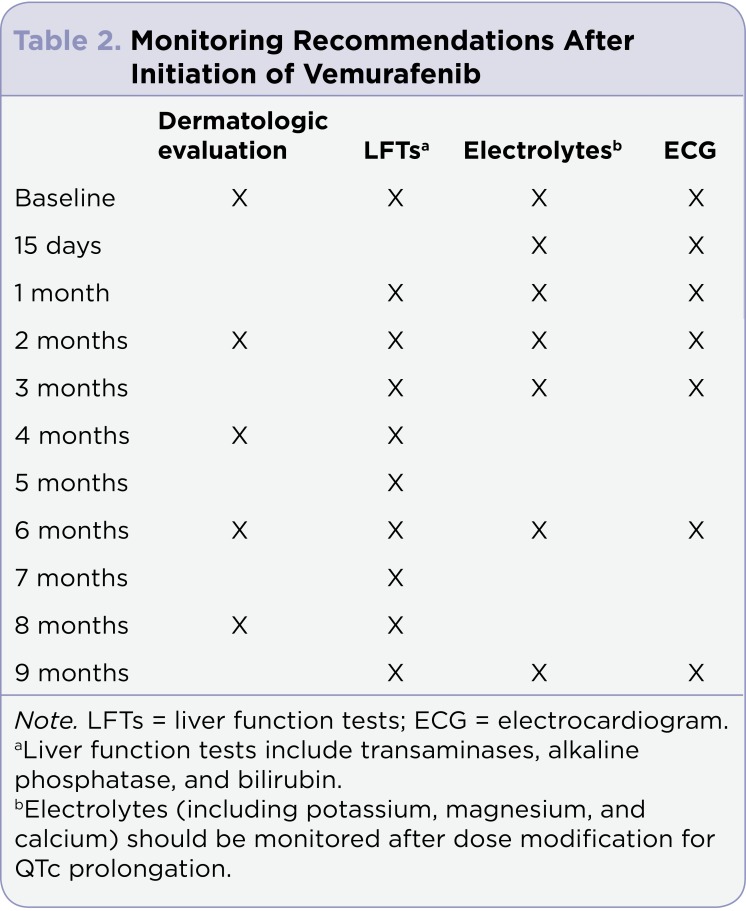
Monitoring Recommendations After Initiation of Vemurafenib

Vemurafenib does not require a dosing adjustment for mild to moderate renal or hepatic impairment, although it has not been tested in patients with severe renal or hepatic impairment. The major metabolism of vemurafenib is through CYP3A4; therefore, providers must be cautious of the potential for drug-drug interactions with numerous other medications that are inhibitors or inducers of this enzyme system. Coadministration of vemurafenib with CYP1A2 substrates should be avoided, as vemurafenib may increase serum concentrations of these drugs (Lexi-Comp, 2014; Genentech, 2013).

## SUMMARY

Mutated *BRAF* is a new target for the treatment of advanced melanoma and provides an important opportunity for new therapies. Vemurafenib is indicated as an option for first-line therapy in patients with unresectable or metastatic melanoma with *BRAF* V600E mutation. Vemurafenib has shown increases in overall and progression-free survival compared with dacarbazine. Due to the potential for serious adverse effects, patients receiving vemurafenib should be monitored closely, including regular dermatologic evaluations.

Vemurafenib is the first of its kind to target mutated *BRAF* and is already opening the door for other therapies. Further studies will help to clarify the role of vemurafenib in combination with other therapies, such as MEK inhibitors.
